# Spatial Variability and Co-acclimation of Phytoplankton and Bacterioplankton Communities in the Pearl River Estuary, China

**DOI:** 10.3389/fmicb.2018.02503

**Published:** 2018-10-23

**Authors:** Jianming Zhu, Yiguo Hong, Sahib Zada, Zhong Hu, Hui Wang

**Affiliations:** ^1^Department of Biology, College of Science, Shantou University, Shantou, China; ^2^School of Environmental Science and Engineering, Guangzhou University, Guangzhou, China

**Keywords:** phytoplankton, bacterioplankton, co-acclimation, network, estuary

## Abstract

Phytoplankton and bacterioplankton play significant roles in estuarine systems. It is important to demonstrate the spatial variability of bacterial and microalgal communities and understand the co-acclimation of these organisms to different environmental factors. In this study, MiSeq sequencing and morphological identification were applied to analyze the variations in bacterial and microalgal communities in the Pearl River Estuary, respectively. Molecular ecological network analysis was used to investigate the potential interactions between microalgae and bacteria and illustrate the responses of these interactions to environmental gradients. The results revealed that microalgal/bacterial communities in freshwater samples were distinct from those in mesohaline water samples. Microalgae affiliated to the genus *Skeletonema* dominated the mesohaline water phytoplankton communities, while *Melosira* was the more abundant genus in freshwater communities. Actinobacteria, Alphaproteobacteria, Betaproteobacteria, and Acidimicrobiia dominated bacterial communities in freshwater samples, while Gammaproteobacteria, Bacilli, and Synechococcophycideae were more abundant in mesohaline water samples. Tightly correlations were observed between phytoplankton and bacterioplankton. These interactions were regarded to be key factors in shaping the community structures. Further, the KEGG database and PICRUSt were used to predict the functions of bacterioplankton in the process of nitrogen cycling. The results indicated that denitrification could play an important role in nitrogen loss and might alleviate the eutrophication in the Pearl River Estuary. Collectively, the results in this study revealed that substantial changes in phytoplankton and bacterioplankton communities were correlated with the gradients of environmental parameters in the Pearl River Estuary. The results also demonstrated that the interactions between phytoplankton and bacterioplankton were important for these organisms to acclimate to changing environments.

## Introduction

Phytoplankton and bacterioplankton are widely acknowledged as the dominant plankton communities in the oceans and in freshwater (Sarmento and Gasol, 2012). Phytoplankton are the major primary producers in aquatic ecosystems, capturing energy from sunlight and transforming inorganic matter to organic matter, providing approximately half of the global net primary productivity (NPP) (Field et al., 1998). Phytoplankton are regarded to be the foundation of the complex marine food web. Phytoplankton often engage in symbiosis with other organisms (especially bacterioplankton) in aquatic ecosystems (Falkowski, 1994). Bacterioplankton represent approximately a quarter of all biomass in the euphotic zones of aquatic habitats and play an important role in aquatic ecosystems by driving biogeochemical cycles (Falkowski et al., 2008). Bacterioplankton can reprocess approximately half of the oceanic NPP in the so-called “microbial loop” (Azam, 1998). In addition, some groups of bacteria, including *Rhodobacter, Hyphomicrobium*, and *Pseudomonas*, have been found to be capable of mediating nitrogen cycling by either fixing gaseous nitrogen for utilization by phytoplankton or by denitrifying nitrogenous compounds to nitrogen gas (Shapleigh, 2006).

Marine ecosystems are constructed and function based on networks that link every species within a certain range of spatial scales via interactions between different organisms and between organisms and environments. Phytoplankton and bacterioplankton can live in the ‘phycosphere’, a term that describes the region surrounding a phytoplankton cell in which the organic molecules exuded by the cell are enriched, leading to a distinct concentration gradient between the cell surface and the surrounding water (Seymour et al., 2017). The strong ecological coupling between these two groups can greatly influence the symbionts of these organisms, the surrounding environments, and even biogeochemical cycling (Natrah et al., 2014). Various relationships between microalgae and bacteria have been well investigated, such as nutrient exchange, signal transduction and gene transfer (Kouzuma and Watanabe, 2015). For instance, *Sulfitobacter*-related bacteria, members of the Rhodobacteraceae family, can enhance the growth of the diatom *Pseudo-nitzschia multiseries* by converting the diatom-secreted amino acid tryptophan, a carbon source, to the growth-promoting hormone indole-3-acetic acid (IAA) (Amin et al., 2015). In addition, bacteria-phytoplankton interactions occur in the phycosphere at the microscale and have important effects on regional-scale climate regulation and ecosystem-scale processes (Seymour et al., 2017). An excellent example of such a phenomenon is the metabolism of the organic sulfur-containing compound dimethylsulfoniopropionate (DMSP), which is degraded by associated bacteria (e.g., *Roseobacter*) (Newton et al., 2010). DMSP is cleaved to generate dimethyl sulfide (DMS). DMS can escape into the atmosphere, where it contributes to the formation of cloud condensation nuclei and to the backscatter of solar radiation (Curson et al., 2011).

As transitional zones between marine and freshwater environments, estuaries are characterized by strong gradients in environmental variables such as salinity and nutrients as a result of river discharge and near-shore human activities. Environmental heterogeneity influences the species diversity and numerical variability of phytoplankton/bacterioplankton in estuaries (Crump et al., 2004). Eutrophic estuaries offer a model ecosystem to study the responses of phytoplankton and bacterioplankton to gradients in environmental factors and to study the contributions of these organisms to biogeochemical cycling.

The Pearl River (PR) is the largest river that discharges into the South China Sea. Generous amounts of freshwater are delivered to the northern South China Sea by the PR from April to September. In recent years, rapid economic growth and anthropogenic stress from cities surrounding the PR Delta have greatly affected the water quality of the PR Estuary (PRE). Large amounts of domestic, industrial, and agricultural effluents are discharged into the PRE, leading to a major concern regarding water quality. The input of nutrients, especially nitrogen, has resulted in aquatic eutrophication in this estuary in recent years. Eutrophic environments provide adequate substrates for vast biomasses of phytoplankton and bacterioplankton in aquatic systems. Previous studies have shown that nutrients and salinity have a strong influence on phytoplankton communities and abundance, with diatoms dominating the phytoplankton communities (Qiu et al., 2010). Similarly, nutrients and salinity were shown to be the key factors responsible for variations in bacterioplankton distribution across stations in the PRE (Liu et al., 2015). However, little is known about the interactions between phytoplankton and bacterioplankton and about the combined actions of these organisms on biogeochemical cycles in estuarine ecosystems.

By analyzing the phytoplankton and bacterioplankton communities in water samples collected from seven sites in the PRE, this study aims to (1) identify the effects of environmental factors on microbial community structures, (2) reveal the potential co-acclimation of different phytoplankton and bacterioplankton groups to changing environments, and (3) investigate the contribution of these organisms to microorganism-driven nitrogen-cycling in estuarine ecosystems.

## Materials and Methods

### Sampling Sites

A total of seven sites were selected along the PRE: S1, S2 and S3, located in the freshwater part of the estuary; S4, located in the brackish region; and S5, S6 and S7, located in the mesohaline water region (Supplementary Figure S1 and Table 1). The water depth at the PRE stations ranged from 6 to 21 m, and the water samples were taken from the surface layers (0.5 m below the surface) of all seven stations during a summer cruise from 15th August 2016 to 17th August 2016.

**Table 1:** Environmental factor parameters of every sampling site.

Site	Longitude	Latitude	Temperature (°C)	Salinity (%)	pH	NO_3_-N (μM)	NO_2_-N (μM)	NH_4_-N (μM)	DIN (μM)	Turbidity (NTU)
S1	113.5167	23.0333	29.49	0.008	7.08	77.19	34.35	39.74	151.28	43.50
S2	113.6233	22.7900	29.68	0.105	7.04	74.50	39.00	20.26	133.76	33.80
S3	113.6818	22.7102	29.46	0.231	7.43	86.55	20.52	6.55	113.62	26.50
S4	113.7506	22.5233	28.52	0.458	7.73	76.83	13.49	2.88	93.20	14.70
S5	113.7520	22.4260	27.61	1.405	7.64	59.58	18.73	4.14	82.45	5.50
S6	113.7221	22.2574	29.44	0.796	7.87	34.63	10.19	2.77	47.59	9.10
S7	113.7221	21.9935	28.15	1.847	8.56	16.51	4.92	0.40	21.83	5.20

### Sample Collection and Determination of Physical and Chemical Parameters

Aliquots (1 L) of the water for bacterial community analysis were filtered through 0.22-μm-pore-size polycarbonate Isopore filter membranes (Millipore, Bedford, United States). The filters were snap frozen in liquid nitrogen and then stored in a -80°C freezer in the laboratory until DNA extraction. Water samples (1 L) for phytoplankton community analysis were collected from the surface layer at each station using polyethylene bottles, and the samples were fixed immediately with 1.5% (final concentration) Lugol’s iodine [5% (w/v) elemental iodine (I_2_), and 10% (w/v) potassium iodide (KI) in distilled water] for microscopic identification. After the fixed water being settled for 24 h and concentrated to 40 ml by gently removing the supernatant, species identification and cell count were carried out under an inverted microscope in a 1 mL subsample by SCSFRI-CAFS (South China Sea Fisheries Research Institute, Chinese Academy of Fishery Sciences). A YSI Hanna HI 9829 water quality monitoring system (YSI Inc., United States) was employed to measure temperature, salinity (psu), pH, and turbidity (NTU) of the surface water. Nitrite and nitrate levels (μM) were measured by using a modified zinc–cadmium reduction method (MZCRM), as described by Wu J. et al. (2016). The dissolved NH^4+^ (μM) of the water samples was analyzed with methods of oxidized by hypobromite (Wu M. et al., 2016).

### DNA Extraction and MiSeq Sequencing

To determine the bacterial community composition, biological samples filtered through 0.22-μm membranes were used to extract total genomic DNA by using a PowerSoil Kit (MoBio Laboratories, Solana Beach, CA, United States) strictly following the manufacturer’s instruction. High-quality DNA was PCR amplified and sequenced by BGI-Shenzhen (The Beijing Genomics Institute, Shenzhen, Guangdong, China). Sequencing were conducted by Illumina MiSeq sequencing to analyze the V4 region of the 16S rRNA gene using the universal primers 515F (5′-GTGYCAGCMGCCGCGGTAA-3′) and 806R (5′-GGACTACNVGGGTWTCTAAT-3′). The raw data were pre-processed to obtain clean data by removing low-quality sequence reads. Sequence reads with a quality average less than 20, within a 30-bp sliding window, were discarded. The paired-end reads were assembled to tags by using FLASH (minimal overlap length, 15 bp; mismatch ratio of overlapping region ≤0.1) (Magoc and Salzberg, 2011). The tags were clustered into different OTUs with a 97% cutoff, and unique representative sequences for each OTU were selected by USEARCH software (Edgar, 2013). Chimeras were filtered out by using UCHIME and mapping to the GOLD database (version 20110519) (Edgar et al., 2011). Representative sequences of different OTUs were aligned against the Greengenes database (version 201305) for taxonomic classification by using Ribosomal Database Project (RDP) Classifier (version 2.2). All sequence data obtained from this study had been deposited to the public National Center for Biotechnology Information (NCBI) database with the Sequence Read Archive accession number: SRP149206.

### Distribution Analysis of Phytoplankton and Bacterioplankton

Correlations between bacterial populations and environmental variables were determined by canonical correspondence analysis (CCA) at the genus level by the vegan package in R software. The statistical significance of the relationship was assessed by the Monte Carlo permutation test using 999 permutations.

### Reconstruction of the Phytoplankton–Bacterioplankton Network

Spearman’s correlation coefficient was used to calculate the correlations between phytoplankton, bacterioplankton, archaea, and environmental factors. The relative abundances of bacterial OTUs, the values of the physical and chemical parameters and the relative abundances of microalgae in each sample were used to construct the correlation matrix. The matrices of the correlation coefficients and the *p*-values were calculated by using R software with the “psych” library by calculating all possible pairwise combinations of Spearman’s rank correlations between all units. Co-occurrence paired with a correlation coefficient >0.7 or <-0.7 with a *p*-value <0.05 was considered to be significantly correlated. The units were further used for network construction. Network visualizations of correlation matrices were generated in Cytoscape_v3.5.1 (Shannon et al., 2003). Network-Analyzer in Cytoscape was used to describe the properties of the individual nodes of the network and the overall topologies or structures. To discern the relationships between biological modules and environmental factors, the distribution of bacteria/phytoplankton and environmental variables were integrated into the networks.

### Function Prediction and Metabolic Pathway Reconstruction

Phylogenetic Investigation of Communities by Reconstruction of Unobserved States (PICRUSt), a predictive exploratory tool, was combined with Kyoto Encyclopedia of Genes and Genomes (KEGG) ortholog classification to predict functional metagenomes from the 16S rRNA gene datasets of each sample (Langille et al., 2013; Kanehisa et al., 2014). In this study, PICRUSt was used to explore the functional profiles of the bacterial communities according to the online protocol^1^.

## Results and Discussion

This study targeted the horizontal distribution patterns of bacterioplankton and phytoplankton communities along the PRE and the potential function of nitrogen cycling in estuaries. To our knowledge, this study was the first investigation of interactions between bacterioplankton and phytoplankton in the PRE.

### Environmental Factors Controlling the Spatial Variations of Phytoplankton

Similar to other estuaries, freshwater discharge created gradients of salinity and of inorganic and organic nutrients in the PRE (Jochem, 2003). Strong variations in salinity, dissolved inorganic nitrogen (DIN), and turbidity were observed at the different sampling sites in the PRE (Table 1). The trends of the physical and chemical environmental factors did not match the linear trend. For instance, the salinity at site S6, concentrations of NO_2_^-^ and NH_4_^+^ at site S4 were lower, while concentration of NO_3_^-^ at sites S3 and S4 were higher than the values of the same parameters at the adjacent stations (Table 1). This could be caused by the dynamic variation in biogeochemical and physical processes in the PRE (Dong et al., 2004). The sampling area was a zone with circulation currents and was centered in the turbidity maxima created by the river flow and tidal forces. These factors are widely acknowledged to be responsible for the transport and distribution of environmental parameters as well as for water column stability (Wong et al., 1995).

**FIGURE 1:**
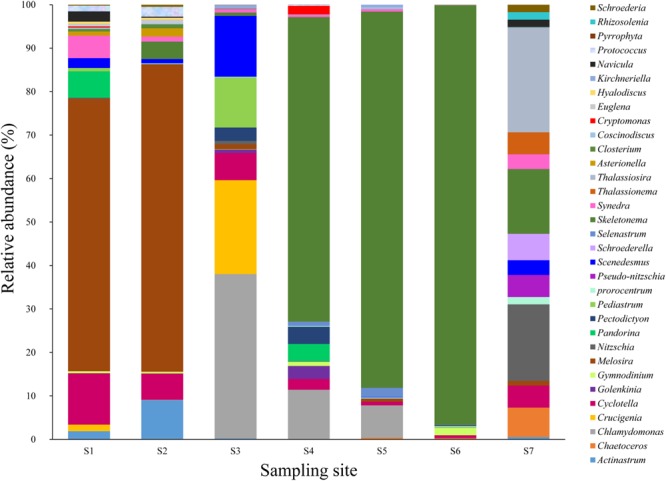
Microalgal composition (at the genus level) at all surface sampling sites. *Y*-axis represents the relative abundance of microalgae. *X*-axis represents the sampling sites.

**FIGURE 2:**
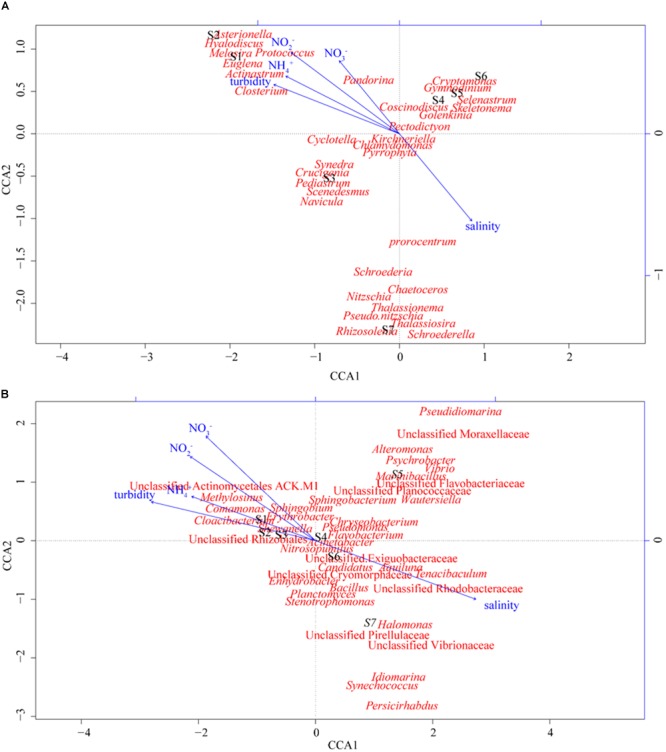
CCA illustrating the relationship between environmental factors and microbial community structure at the genus level at different sampling sites. **(A)** Phytoplankton community and **(B)** bacterioplankton community.

More than 80 phytoplankton genera were identified by microscopic identification in this study (Figure 1). Significant variations in phytoplankton communities were observed along the PRE. Diatoms dominated the phytoplankton communities at the freshwater sites S1 and S2 (representative genus *Melosira*) and the mesohaline water sites S4, S5, S6, and S7 (representative genera *Skeletonema, Nitzschia*, and *Thalassiosira*), while chlorophytes dominated site S3 (representative genera *Chlamydomonas* and *Crucigenia*) (Figure 1). Diatoms were also found to be the predominant phytoplankton group in Tagus Estuary (Gameiro et al., 2007). Previous studies have shown that diatoms are the dominant group of phytoplankton and contribute to almost 40% of the primary productivity in marine ecosystems (Rabosky and Sorhannus, 2009). This results were similar with previous study that genus *Melosira* was often dominant the upper part of the PRE, while genus *Skeletonema* was the dominant in the lower estuary (Huang et al., 2004). The composition and dominant species of phytoplankton were highly sensitive to environmental changes. Several studies have shown that environmental factors, such as salinity and nutrient concentration, have significant effects on the compositions of phytoplankton communities (Suikkanen et al., 2007). Salinity is thought to be the primary factor responsible for shaping phytoplankton community structures in estuaries (Muylaert et al., 2000). DIN is also an important factor influencing the diversities of phytoplankton communities (Suikkanen et al., 2007). Other physical forces, such as wind, human activities, zooplankton grazing, and water current also could regulate the phytoplankton community in the estuary (Harrison et al., 2008). For instance, the station S3 was at the junction of distributaries, the phytoplankton composition in S3 was different from nearby stations S2 and S4. In addition, chlorophytes dominated station S3 were reported to be sensitive to the salinity changes (Li et al., 2014), which might result in the decrease of chlorophytes at station S4. CCA was performed to discern possible associations between environmental factors and distribution of phytoplankton (Figure 2A). The sum of all the canonical eigenvalues indicated that 89.26% of the total variation in the phytoplankton communities could be explained by environmental variations. The first two CCA axes explained 72.08% of the total variance in phytoplankton composition. Salinity appeared to be a significant parameter influencing the distribution of phytoplankton along with DIN and turbidity. In this study, steep gradients of DIN and salinity along the PRE greatly affected the distribution of the dominant species of phytoplankton. *Melosira* was the dominant genus at the freshwater sites, while *Chlamydomonas* and *Skeletonema* were dominant at the mesohaline water sites (Figure 1). From stations S4 to S6, the diatom *Skeletonema* gradually developed a growth advantage as the relative abundance of *Chlamydomonas* decreased. This observation might be associated with increased salinity. *Skeletonema* is a genus of typical salt phytoplankton, which can grow in saline (1.3–3.6%) growth environments. *Thalassiosira* affiliated with the diatom that dominated the phytoplankton community at station S7, has been acknowledged to be capable of living in both sea water and fresh water (Hasle and Lange, 1989). Microalgae in this genus might have a competitive advantage over *Skeletonema* under conditions of high salinity and low DIN at station S7.

The nitrate (NO_3_^-^) concentrations (16.51∼86.55 μM) were greater than those of nitrite (NO_2_^-^) (4.92∼39.00 μM) and ammonium (NH_4_^+^) (0.90∼39.74 μM) at all the study sites. The highest NO_3_^-^ concentration was observed at site S5, with a value of 86.55 μM (Table 1). Although NO_3_^-^ was the dominant form of DIN in the PRE, NH_4_^+^ was observed to significantly influence the phytoplankton diversity (*R*^2^ = 0.8012, *p* < 0.05), which was revealed by the analysis based on the envfit function module of the vegan package in R. Previous studies showed that NH_4_^+^, not NO_3_^-^, is usually the primary form of nitrogen taken up by phytoplankton (Collos et al., 2004).

The concentration of DIN at the mesohaline water sites was much less than that at the freshwater sites. The concentration of DIN was 2.8 times higher at the freshwater site S2 than that at the mesohaline water site S6. However, the abundance of phytoplankton at the mesohaline water site S6 (∼4.85 × 10^5^ cells/L) was more than that at the freshwater site S2 (∼1.00 × 10^5^ cells/L), which could be caused by the variation in the turbidity gradients of the two regions. The turbidity of water varied from 9.10 NTU (S6) to 33.80 NTU (S2). High turbidity could result in severe light scattering and could reduce light penetration, consequently inhibiting the growth and photosynthesis of phytoplankton in estuaries (Oliver et al., 2010). Although nutrients at the freshwater sites of the estuary were plentiful, high concentrations of suspended sediments could lead to low concentrations of phytoplankton. Similar phenomena were also detected at the Chesapeake Bay and the Delaware Estuary by measuring the distributions of turbidity, nutrients, and phytoplankton across the salinity gradients of these estuaries (Fisher et al., 1988).

### Spatial Distribution of Bacterioplankton Communities

**FIGURE 3:**
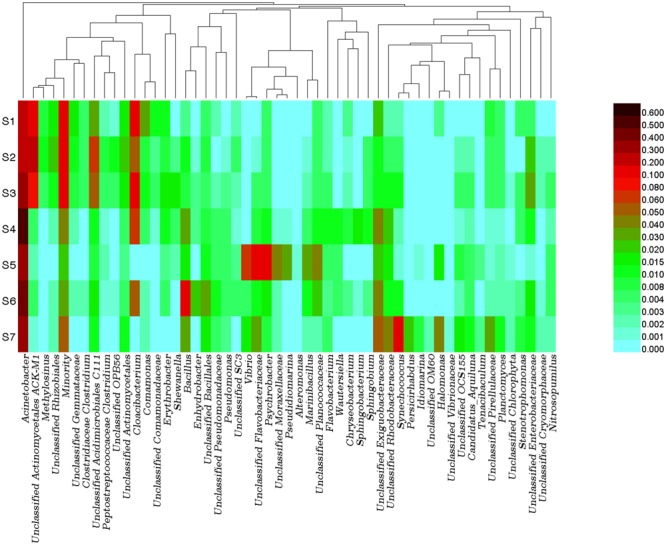
Bacterial community compositions in water from sampling sites. Relative abundance of the dominant bacterial genus (threshold value: at least one site with bacterial percentage greater than 1%). The color scale indicated the relative abundances of bacterioplankton at each station.

In total, more than 41 bacterial phyla were detected at all sites of the PRE. Similar to previous study, the phylum proteobacteria was the dominant bacterial group in the PRE (Wu et al., 2004) and Weser estuary (Selje and Simon, 2003). Although Proteobacteria dominated the bacterioplankton communities in almost all the samples, significant variations in bacterioplankton communities at the phylum level were observed along the salinity gradient. For instance, the relative abundances of Actinobacteria and Bacteroidetes were dramatically higher at the freshwater sites S1, S2, and S3, while those of Firmicutes was more abundant at the mesohaline water sites S4, S5, S6, and S7 (Supplementary Figure S2). To analyze the distributions of bacterioplankton in detail, a heatmap was generated at the genus level based on OTU relative abundance (Figure 3). Consistent with the results obtained for bacterioplankton at the phylum level, some groups affiliated with Proteobacteria (e.g., *Acinetobacter*) were abundant at all sites. Upon comparing the differences between the dominant bacterioplankton at the freshwater and mesohaline water sites, significant spatial variation in bacterioplankton communities was observed. For instance, *Synechococcus, Vibrio, Bacillus*, an unclassified group of the Exiguobacteraceae family, and an unclassified group of the Rhodobacteraceae family were abundant in the mesohaline water samples, while *Cloacibacterium, Comamonas*, an unclassified group of Actinomycetales ACK-M1, an unclassified group of Acidimicrobiales C111, and an unclassified group of the order Rhizobiales were abundant in the freshwater samples from the PRE. Bacterial communities consistently exhibit significant spatial differences owing to gradients of salinity and nutrients. CCA was also performed to discern possible associations between environmental factors and distributions of bacterioplankton, with environmental factors as explanatory variables (Figure 2B). The sum of all the canonical eigenvalues indicated that 93.8% of the total variation can be explained by environmental variations. The first two CCA axes explained 71.0% of the total variance in phytoplankton composition. Salinity had a significantly different (*p* < 0.001) effect on the distribution of bacterioplankton than that of DIN and turbidity, as determined by the envfit function (number of permutations: 999). The results of this study also suggested that bacterioplankton communities were positively associated with nutrient and salinity gradient. Similar to the results of previous studies on estuaries, the compositions of bacterioplankton were significantly different between the freshwater and mesohaline water sites of the PRE (Zhang et al., 2006). The results in this study revealed that salinity (*R*^2^ = 0.94, *p* < 0.001) was the dominant factor responsible for the diversity of bacterioplankton along the PRE.

Flavobacteria are most abundant in both mesohaline water and freshwater regions (Alonso et al., 2007). However, different groups of Flavobacteriales were dominant at sampling sites with different salinity concentrations. For instance, *Cloacibacterium* dominated the bacterioplankton community at the freshwater sites, while an unclassified group of Flavobacteriaceae was dominant at the mesohaline water sites. Actinobacteria were thought to play an important role in the process of organic matter decomposition and mineralization (Anesio et al., 2003). These bacteria were significantly (*p* < 0.05) more abundant at the freshwater sites than at the mesohaline water sites. Cyanobacteria, such as *Synechococcus*, which play an important role in nitrogen fixation, were more abundant at the mesohaline water sites. *Synechococcus* has a negative relationship with DIN and had high relative abundance at the mesohaline water sites, with the maximum value at the oligotrophic site S7. *Synechococcus* species consistently exhibit a competitive advantage over other larger photoautotrophs at lower nutrient concentrations due to the large surface/volume ratios of these bacteria (Partensky et al., 1999). The mesohaline water sites, with high salinity and low DIN, provided suitable environments for the growth of *Synechococcus*. The result of this study also revealed that *Bacilli* and *Vibrio* were abundant at the mesohaline water sites with low DIN. Bacteria from these two groups have been acknowledged to be heterotrophic nitrogen-fixing bacteria in low-nitrogen environments (Shieh et al., 1988).

### The Potential Relationships Between Bacteria, Phytoplankton and DIN

In addition to environmental factors, interactions between different planktons also play an essential role in shaping microbial distribution patterns. Microorganisms (including viruses, bacteria, archaea, and protists) do not exist in isolation in natural environments but form complex ecological interaction webs. Marine ecosystems are composed of networks that connect different organisms across a range of scales and are maintained by species-species interactions, including mutualism, competition, and parasitism (Estes et al., 2011). High-throughput sequencing of taxonomic markers, such as the 16S rRNA gene, has revealed diverse microbial components in specific niches. Computing the pairwise correlation between each unit is one of the most straightforward methods to predict microbial interaction networks by using a correlation coefficient such as Pearson’s product-moment correlation coefficient or Spearman’s nonparametric rank correlation coefficient. Correlation-based network analysis using taxonomic data could uncover potential interactions between microbes in a complex community.

**FIGURE 4:**
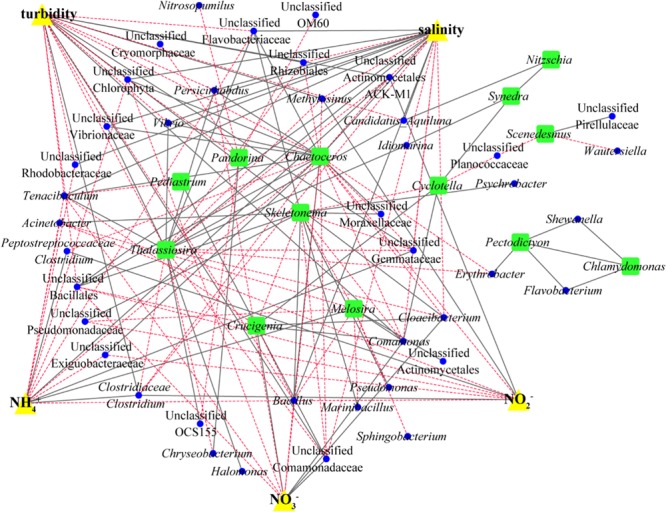
Network analysis reveals trends in plant-adapted and beneficial bacteria. A Spearman correlation network was determined for taxa with average relative abundance greater than 1%. Significant positive correlations (*P* < 0.05) (solid gray lines) and negative correlations (dotted red lines) between microalgae (green dots) and bacterial OTUs (blue dots). Correlations between different phytoplankton groups are also shown as well as correlations between different bacterial groups.

In this study, a correlation-based ecological network was constructed to identify the relationship between microalgae and bacteria by using the relative abundances of bacteria and microalgae in each sample. To further explore the effects of environmental factors on microbial association, environmental factors were also included in the network (Figure 4). The genus-level network consisted of 59 nodes and 165 edges. Salinity and turbidity were seen to be most closely associated with different bacterial taxa; both were seen to be correlated with 21 bacterial taxa. Environmental factors showed tight connections with phytoplankton and bacterioplankton, which suggested that environmental factors (mainly salinity and dissolved inorganic nitrogen in this study) maintained close relationships with these organisms and played an important role in shaping the community structures. In addition, the interactions between phytoplankton and bacterioplankton were also important in shaping the community structures. Some bacterial phylotypes only showed positive or negative relationship with single phytoplankton, which indicated that these bacterioplankton maintained symbiotic relationships only with specific phytoplankton. For example, bacteria in genus *Halomonas* just showed positive relationship with phytoplankton genus *Thalassiosira*. Meanwhile, some bacterial groups presented complex relationship with different phytoplankton. This might be attributed to the similar biochemical environments that the host algae could provide for the symbiotic bacteria. For instance, bacteria in genus *Flavobacterium* showed positive correlations with two different phytoplankton phylotypes, *Pectodictyon* and *Chlamydomonas*. Furthermore, some bacterial phylotypes showed negative relationship with one kind of phytoplankton, while showed positive relationship with the other one. This resulted a screening effect on the parasitic bacterioplankton by different phytoplankton. For example, *Comamonas* showed positive correlation with the diatom *Melosira*, while showed negative correlation with *Skeletonema*. Focusing on identified cluster structures of the microbiome network based on topology could provide us with invaluable insight into complex interactions and patterns. The nods connected by lines in the network could be divided into some specific identifying clusters (IC). The identified clusters were composed of a group of interconnected units. These identified clusters could be separated from the whole network as subnetworks by using the Cytocluster tool in Cytoscape software. For instance, the identified cluster IC1 consisted of five units, namely, *Flavobacterium, Shewanella, Erythrobacter, Chlamydomonas*, and *Pectodictyon* (Supplementary Figure S3). This cluster revealed the potential ecological relationships among the five factors. Some of these co-occurrences indicated that components of the subnetwork performed similar or complementary functions, while others indicated that the members in the subnetwork shared or preferred similar environmental conditions. *Pectodictyon* and *Chlamydomonas* were both affiliated with green algae and the relative abundances of these organisms were positively correlated, which indicated that these organisms performed similar ecological roles. Considering that these two organisms are ecologically identical, other taxa could respond to both green algae similarly by creating shared neighbors. In fact, *Pectodictyon* and *Chlamydomonas* had two shared neighborhoods, namely, *Flavobacterium* and *Shewanella*. As the characteristic bacteria in the process of algal blooming, Flavobacteria are typically abundant during the decay of phytoplankton blooms and exhibit algicidal activity (Buchan et al., 2014). In addition, phytoplankton can produce large amounts of transparent exopolymer particles (TEPs) to stimulate the growth of Flavobacteriales in coastal water (Taylor et al., 2014). *Shewanella* species have also been shown to be capable of producing active algicidal substances (Pokrzywinski et al., 2012). The subnetwork IC2 is divided into two parts by gray solid lines. This division generated two centers, namely, *Melosira* and *Skeletonema* (Supplementary Figure S4). *Skeletonema* and *Melosira* were the two main groups of diatoms in the PRE, but these organisms showed different distributions along the PRE. *Skeletonema* was the dominant phytoplankton at the mesohaline water sites, while *Melosira* dominated the freshwater sites. Bacterial phylotypes associated with diatoms are believed to assist microalgae in adapting to changing environments. In fact, Dittami et al. (2016) demonstrated that *Ectocarpus*, a genus of small filamentous brown algae, could grow in high and low salinities depending on the microbes it hosts. In addition, the genus *Comamonas* exhibited distinct positive correlation with the diatom *Melosira*, while the genus *Pseudomonas* showed positive correlation with *Skeletonema*. *Comamonas* have been shown to be denitrifying bacteria (Gumaelius et al., 2001). In the high-DIN region, *Comamonas* might utilize microalgal secretions to grow and denitrify NO_3_^-^ to NH_4_^+^ for the growth of microalgae. In contrast, *Pseudomonas* exhibit nitrogen fixation (Yu et al., 2011). In the low-DIN region, at the mesohaline water sites, *Pseudomonas* might perform nitrogen fixation and exist in symbiosis with *Skeletonema*. Bacteria affiliated in the family of Flavobacteria and Sphingobacteria in the phylum Bacteroidetes were also found to be associated with diatoms in the subnetwork IC2. Flavobacteria had been acknowledged as a model for the study of bacterioplankton–phytoplankton interactions, in view of their capability of transforming the phytoplankton-derived higher molecular weight macromolecules including polysaccharides and proteins to low molecular weight molecules (Buchan et al., 2014). The importance of Sphingobacteria during phytoplankton blooms was also revealed in previous study (Qu et al., 2015). Bacteria in this family could degrade complex organic macromolecules and mediate the biogeochemical recycling of nitrogen and phosphorus (Qu et al., 2015).

Interestingly, at the mesohaline water sites, the bacterial phylotype *Bacillus* and the microalgae *Skeletonema* were abundant and exhibited positive correlation (IC3, Supplementary Figure S5). *Bacillus* was shown to contain all the genes associated with the pathways of assimilatory nitrate reduction (*narB, nasA, nirA*) and dissimilatory nitrate reduction (*narG, narH, nirD*), which can catalyze NO_3_^-^ to generate NH_4_^+^. It is believed that phytoplankton assimilate NH_4_^+^ and then synthesize amino acids directly from the intracellular keto acid. In most cases, even when the oxidation of nitrogen sources (such as NO_3_^-^ and NO_2_^-^) is more abundant, phytoplankton tend to use reduced nitrogen (NH_4_^+^ and urea) (Fan et al., 2003). Therefore, *Bacillus* might generate ammonium by reduction of nitrate and nitrite to promote the growth of *Skeletonema*.

Correlation networks are important for visualizing and analyzing statistical associations among microorganisms and between microbes and environmental factors. In addition, identified clusters of the correlation network could suggest shared niches, symbiosis and other relationships. Our network analysis revealed complex relationships among phytoplankton, bacterioplankton, and environmental factors and revealed important details regarding the rules of community assembly in the PRE. The results of this study revealed that the assemblage patterns of the microbial communities were non-random and revealed interactions between phytoplankton and bacterioplankton. In the correlation networks, some phytoplankton showed positive correlation with more than one bacterial phylotype. This observation indicated that the bacterial phylotypes of the phycosphere might share similar ecological niches with the support of phytoplankton or by performing complementary functions to each other. Some bacterial phylotypes also showed positive correlation with more than one phytoplankton. This nonspecific species–species correlation between phytoplankton and bacterioplankton was the foundation of the network. In addition, the results showed that the different identified clusters, including phytoplankton and bacterioplankton, were influenced by different environmental factors. This result revealed the co-acclimation of phytoplankton and bacterioplankton to different environmental factors such as salinity and nitrogen.

### Microbial Nitrogen Cycling in the PRE: From Phylogeny to Genes

**FIGURE 5:**
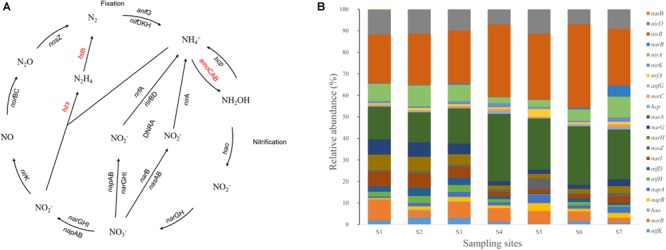
Predicted nitrogen cycling pathway modified from KEGG **(A)**. Red-colored genes were not predicted by the PICRUSt or were undetected in the indicated samples. Gene relative abundance in nutrient-cycling processes **(B)**.

Nitrogen cycling is the key process of the biogeochemical cycle and energy flow in marine ecosystems and is important for the maintenance and restoration of ecological balance. However, excessive DIN might accelerate eutrophication and global climate change, resulting in ecological and environmental problems, such as water hypoxia, algal blooms, biodiversity decrease, and decreased fishery productivity (Rabalais et al., 2009). Microorganisms are the main drivers of the nitrogen cycle in estuarine ecosystems. The functions of the different OTUs and the proportions of the functional bacterial groups participating in each step of the nitrogen cycle were predicted by using KEGG and PICRUSt. The contribution and quantity of the functional genes of microbes in nitrogen fixation, ammonification, nitrification, denitrification, dissimilatory nitrate reduction to ammonium, and anammox in the PRE were investigated in this study (Figure 5). Significant differences were showed between the freshwater and mesohaline sites. The relative abundances of genes *nirB* and *nasA* are predominant in all stations. And the relative abundance of genes *nir*B (*P* < 0.05) and *nas*A (*P* < 0.01) in the mesohaline stations S5, S6, S7, and the brackish station S4 were higher than those in freshwater stations S1, S2, and S3. In contrast, the relative abundances of the genes *nar*G (*P* < 0.01), *nar*H (*P* < 0.01), and *nar*I (*P* < 0.01) are higher in freshwater stations.

Denitrification is an important way to alleviate the eutrophication of water bodies. Globally, approximately 50% of the total N entering estuaries is removed by denitrification (Howarth et al., 1996). Therefore, it is important to understand the mechanism of denitrification in estuarine areas, leading to the self-purification of estuaries. The results of this study showed that denitrification is the dominant process for nitrogen loss from the surface waters of the PRE, while the relative abundance of the amo genes (which catalyzes oxidation) were negligible (Figures 5A,B).

By analyzing functional genes, we determined that different bacterial phylotypes were responsible for different functional genes at the same station. This result indicated that bacterial communities cooperate to conduct nitrogen metabolism in natural environments. In addition, bacterial phylotypes possessing the same functional genes were present at different sites (Supplementary Figure S6). For example, the main bacterial phylotype possessing the gene *hao*, a key gene associated with nitrification, was ZA3312c (a group of SAR406) at station S7 and *Methylocaldum* at station S1, while it was present as an unclassified bacterial group of the order Chlorobi OPB56 at stations S6, S5, S4, S3, and S2. Similarly, a group of bacteria possessing the genes *narG, narH* and, such as *Halomonas, Bacillus, Psychrobacter*, dominated the mesohaline water and *narI* sites, while an unclassified bacterial group of the family ACK-M1 was abundant at the freshwater sites. Bacteria harboring nitrogen-fixation-associated genes (*nifH, nifD*, and *nifK*) also exhibited similar patterns. *Yersinia, Pseudomonas*, and *Methylosinus* have been identified to be the main functional bacterial phylotypes for nitrogen fixation (Toukdarian and Lidstrom, 1984) but were observed to be responsible for nitrogen fixation at different sites. For example, *Yersinia* mainly contributed to nitrogen fixation at sites S2, S3, S4, S6, and S7, while *Pseudomonas* and *Methylosinus* were responsible for the process at sites S5 and S1, respectively.

Some bacteria containing several genes associated with nitrogen-cycling processes might play important roles in nitrogen cycling. An unclassified bacterial group of the family Rhodobacteraceae contained the genes *nirB, nirD, nirK, narG, nasA, narl, norC, napA, nifD, nifK, nifH, norB*, and *hcp* and was the dominant bacterial phylotype in the mesohaline water at site S7. Bacteria affiliated with Rhodobacteraceae have been acknowledged to play significant roles in the nitrogen cycle (Coates and Wyman, 2017). Previous studies have demonstrated that *Roseobacter*, affiliated with Rhodobacteraceae, can rapidly assimilate ammonium and use dissolved organic nitrogen (DON) as a N source (Mayali et al., 2014). In addition, some functional genes were found to be abundant at all sampling sites and were consistently harbored by a single bacterial phylotype. This finding indicated that the bacterial phylotype might play an irreplaceable role in catalyzing the metabolic step. For instance, an unclassified bacterial group of the family Hyphomicrobiaceae contributed the gene *anfG*, which is associated with the catalysis of nitrogen to ammonia at all sites. A previous study showed that some groups of the family Hyphomicrobiaceae had the ability to fix nitrogen (Rivas et al., 2003).

## Conclusion

Based on high-throughput sequencing data analysis, CCA and network construction, this study demonstrated the co-succession of bacterial communities and phytoplankton communities as well as the relationships of these communities with changing environmental factors. Our results demonstrated that DIN and salinity can greatly influence the distribution of phytoplankton and bacterioplankton. In addition, the co-occurrence patterns and the relationships of phytoplankton and bacterioplankton were seen to be responsible for the capacity of these organisms to co-acclimate to changing environmental factors. Bacteria can perform several functions in the nitrogen cycle, especially in denitrification, in the PRE. Overall, phytoplankton and bacterioplankton co-acclimate to environmental changes and play an important role in biogeochemical cycling of nitrogen by means of network interactions in estuarine ecosystems. However, microbial interactions are complex, and little is known about the metabolic characteristics of these interactions. It is challenging to understand the interactions between phytoplankton and bacterioplankton at the metabolic levels in constantly changing environments. Metagenomics, metatranscriptomics and other omic tools are necessary for further investigation of these complex interactions.

## Availability of Data and Materials

Raw sequencing data with SRA accession number SRP149206 were available at Sequence Read Archive at the website of National Center for Biotechnology Information (NCBI).

## Author Contributions

JZ, YH, ZH, and HW designed the experiments. JZ and HW performed the experiments. JZ and HW collected and analyzed the data. JZ, SZ, and HW wrote the draft of the article. JZ, YH, SZ, ZH, and HW revised the draft of the article.

## Conflict of Interest Statement

The authors declare that the research was conducted in the absence of any commercial or financial relationships that could be construed as a potential conflict of interest. The reviewer AC and handling Editor declared their shared affiliation.

## Footnotes

1 http://picrust.github.io/picrust/

## References

[B1] Alonso C. Warnecke F. Amann R. Pernthaler J. (2007). High local and global diversity of Flavobacteria in marine plankton. *Environ. Microbiol.* 9 1253–1266. 10.1111/j.1462-2920.2007.01244.x 17472638

[B2] Amin S. A. Hmelo L. R. van Tol H. M. Durham B. P. Carlson L. T. Heal K. R. (2015). Interaction and signalling between a cosmopolitan phytoplankton and associated bacteria. *Nature* 522 98–101. 10.1038/nature14488 26017307

[B3] Anesio A. M. Abreu P. C. Biddanda B. A. (2003). The role of free and attached microorganisms in the decomposition of estuarine macrophyte detritus. *Estuar. Coast. Shelf Sci.* 56 197–201. 10.1016/s0272-7714(02)00152-x

[B4] Azam F. (1998). Microbial control of oceanic carbon flux: the plot thickens. *Science* 280 694–696. 10.1126/science.280.5364.694

[B5] Buchan A. LeCleir G. R. Gulvik C. A. Gonzalez J. M. (2014). Master recyclers: features and functions of bacteria associated with phytoplankton blooms. *Nat. Rev. Microbiol.* 12 686–698. 10.1038/nrmicro3326 25134618

[B6] Coates C. J. Wyman M. (2017). A denitrifying community associated with a major, marine nitrogen fixer. *Environ. Microbiol.* 19 4978–4992. 10.1111/1462-2920.14007 29194965

[B7] Collos Y. Gagne C. Laabir M. Vaquer A. Cecchi P. Souchu P. (2004). Nitrogenous nutrition of *Alexandrium catenella* (Dinophyceae) in cultures and in Thau lagoon, southern France. *J. Phycol.* 40 96–103. 10.1046/j.1529-8817.2004.03034

[B8] Crump B. C. Hopkinson C. S. Sogin M. L. Hobbie J. E. (2004). Microbial biogeography along an estuarine salinity gradient: combined influences of bacterial growth and residence time. *Appl. Environ. Microbiol.* 70 1494–1505. 10.1128/aem.70.3.1494-1505.2004 15006771 PMC365029

[B9] Curson A. R. J. Todd J. D. Sullivan M. J. Johnston A. W. B. (2011). Catabolism of dimethylsulphoniopropionate: microorganisms, enzymes and genes. *Nat. Rev. Microbiol.* 9 849–859. 10.1038/nrmicro2653 21986900

[B10] Dittami S. M. Duboscq-Bidot L. Perennou M. Gobet A. Corre E. Boyen C. (2016). Host-microbe interactions as a driver of acclimation to salinity gradients in brown algal cultures. *ISME J.* 10 51–63. 10.1038/ismej.2015.104 26114888 PMC4681850

[B11] Dong L. Su J. Wong L. Cao Z. Chen J. (2004). Seasonal variation and dynamics of the Pearl River plume. *Continent. Shelf Res.* 24 1761–1777. 10.1016/j.csr.2004.06.006 28823423

[B12] Edgar R. C. (2013). UPARSE: highly accurate OTU sequences from microbial amplicon reads. *Nat. Methods* 10 996–998. 10.1038/nmeth.2604 23955772

[B13] Edgar R. C. Haas B. J. Clemente J. C. Quince C. Knight R. (2011). UCHIME improves sensitivity and speed of chimera detection. *Bioinformatics* 27 2194–2200. 10.1093/bioinformatics/btr381 21700674 PMC3150044

[B14] Estes J. A. Terborgh J. Brashares J. S. Power M. E. Berger J. Bond W. J. (2011). Trophic downgrading of planet earth. *Science* 333 301–306. 10.1126/science.1205106 21764740

[B15] Falkowski P. G. (1994). The role of phytoplankton photosynthesis in global biogeochemical cycles. *Photosynth. Res.* 39 235–258. 10.1007/bf00014586 24311124

[B16] Falkowski P. G. Fenchel T. Delong E. F. (2008). The microbial engines that drive Earth’s biogeochemical cycles. *Science* 320 1034–1039. 10.1126/science.1153213 18497287

[B17] Fan C. Glibert P. M. Alexander J. Lomas M. W. (2003). Characterization of urease activity in three marine phytoplankton species, *Aureococcus anophagefferens, Prorocentrum minimum*, and *Thalassiosira weissflogii*. *Mar. Biol.* 142 949–958. 10.1007/s00227-003-1017-8

[B18] Field C. B. Behrenfeld M. J. Randerson J. T. Falkowski P. (1998). Primary production of the biosphere: integrating terrestrial and oceanic components. *Science* 281 237–240. 10.1126/science.281.5374.237 9657713

[B19] Fisher T. R. Harding L. W. Stanley D. W. Ward L. G. (1988). Phytoplankton, nutrients, and turbidity in the Chesapeake, Delaware, and Hudson estuaries. *Estuar. Coast. Shelf Sci.* 27 61–93. 10.1016/0272-7714(88)90032-7

[B20] Gameiro C. Cartaxana P. Brotas V. (2007). Environmental drivers of phytoplankton distribution and composition in Tagus Estuary, Portugal. *Estuar. Coast. Shelf Sci.* 75 21–34. 10.1016/j.ecss.2007.05.014

[B21] Gumaelius L. Magnusson G. Pettersson B. Dalhammar G. (2001). *Comamonas denitrificans* sp. nov., an efficient denitrifying bacterium isolated from activated sludge. *Int. J. Syst. Evol. Microbiol.* 51(Pt 3) 999–1006. 10.1099/00207713-51-3-999 11411726

[B22] Harrison P. J. Yin K. Lee J. H. W. Gan J. Liu H. (2008). Physical–biological coupling in the Pearl River Estuary. *Continent. Shelf Res.* 28 1405–1415. 10.1016/j.csr.2007.02.011

[B23] Hasle G. R. Lange C. B. (1989). Freshwater and brackish water Thalassiosira (Bacillariophyceae): taxa with tangentially undulated valves. *Phycologia* 28 120–135. 10.2216/i0031-8884-28-1-120.1

[B24] Howarth R. W. Billen G. Swaney D. Townsend A. Jaworski N. Lajtha K. (1996). “Regional nitrogen budgets and riverine N & P fluxes for the drainages to the North Atlantic Ocean: natural and human influences,” in *Nitrogen Cycling in the North Atlantic Ocean and its Watersheds* ed. Howarth R. W. (Dordrecht: Springer) 75–139. 10.1007/978-94-009-1776-7_3

[B25] Huang L. Jian W. Song X. Huang X. Liu S. Qian P. (2004). Species diversity and distribution for phytoplankton of the Pearl River estuary during rainy and dry seasons. *Mar. Pollut. Bull.* 49 588–596. 10.1016/j.marpolbul.2004.03.015 15476837

[B26] Jochem F. J. (2003). Photo- and heterotrophic pico- and nanoplankton in the Mississippi River plume: distribution and grazing activity. *J. Plankt. Res.* 25 1201–1214. 10.1093/plankt/fbg087

[B27] Kanehisa M. Goto S. Sato Y. Kawashima M. Furumichi M. Tanabe M. (2014). Data, information, knowledge and principle: back to metabolism in KEGG. *Nucleic Acids Res.* 42 D199–D205. 10.1093/nar/gkt1076 24214961 PMC3965122

[B28] Kouzuma A. Watanabe K. (2015). Exploring the potential of algae/bacteria interactions. *Curr. Opin. Biotechnol.* 33 125–129. 10.1016/j.copbio.2015.02.007 25744715

[B29] Langille M. G. I. Zaneveld J. Caporaso J. G. McDonald D. Knights D. Reyes J. A. (2013). Predictive functional profiling of microbial communities using 16S rRNA marker gene sequences. *Nat. Biotechnol.* 31 814–821. 10.1038/nbt.2676 23975157 PMC3819121

[B30] Li G. Lin Q. Lin J. Song X. Tan Y. Huang L. (2014). Environmental gradients regulate the spatial variations of phytoplankton biomass and community structure in surface water of the Pearl River estuary. *Acta Ecol. Sin.* 34 129–133. 10.1016/j.chnaes.2014.01.002

[B31] Liu J. Fu B. Yang H. Zhao M. He B. Zhang X. (2015). Phylogenetic shifts of bacterioplankton community composition along the Pearl Estuary: the potential impact of hypoxia and nutrients. *Front. Microbiol.* 6:64. 10.3389/fmicb.2015.00064 25713564 PMC4322608

[B32] Magoc T. Salzberg S. L. (2011). FLASH: fast length adjustment of short reads to improve genome assemblies. *Bioinformatics* 27 2957–2963. 10.1093/bioinformatics/btr507 21903629 PMC3198573

[B33] Mayali X. Weber P. K. Mabery S. Pett-Ridge J. (2014). Phylogenetic patterns in the microbial response to resource availability: amino acid incorporation in san Francisco bay. *PLoS One* 9:e95842. 10.1371/journal.pone.0095842 24752604 PMC3994146

[B34] Muylaert K. Sabbe K. Vyverman W. (2000). Spatial and Temporal Dynamics of Phytoplankton Communities in a Freshwater Tidal Estuary (Schelde, Belgium). *Estuar. Coast. Shelf Sci.* 50 673–687. 10.1006/ecss.2000.0590

[B35] Natrah F. M. I. Bossier P. Sorgeloos P. Yusoff F. M. Defoirdt T. (2014). Significance of microalgal- bacterial interactions for aquaculture. *Rev. Aquacult.* 6 48–61. 10.1111/raq.12024

[B36] Newton R. J. Griffin L. E. Bowles K. M. Meile C. Gifford S. Givens C. E. (2010). Genome characteristics of a generalist marine bacterial lineage. *ISME J.* 4 784–798. 10.1038/ismej.2009.150 20072162

[B37] Oliver R. L. Mitrovic S. M. Rees C. (2010). Influence of salinity on light conditions and phytoplankton growth in a turbid river. *River Res. Appl.* 26 894–903. 10.1002/rra.1309

[B38] Partensky F. Blanchot J. Vaulot D. (1999). Differential distribution and ecology of Prochlorococcus and Synechococcus in oceanic waters: a review. *Bull. Institut. Oceanogr. Mon. Num. Spec.* 19 457–476.

[B39] Pokrzywinski K. L. Place A. R. Warner M. E. Coyne K. J. (2012). Investigation of the algicidal exudate produced by *Shewanella* sp. *IRI-*160 and its effect on dinoflagellates. *Harmful Algae* 19(Suppl. C) 23–29. 10.1016/j.hal.2012.05.002

[B40] Qiu D. Huang L. Zhang J. Lin S. (2010). Phytoplankton dynamics in and near the highly eutrophic Pearl River Estuary, South China Sea. *Continent. Shelf Res.* 30 177–186. 10.1016/j.csr.2009.10.015 21316714

[B41] Qu J. Zhang Q. Zhang N. Shen L. Liu P. (2015). “Microbial community diversity in water and sediment of an eutrophic lake during harmful algal bloom using MiSeq illumina technology,” in *Proceedings of the International Conference on Advances in Environment Research* Paris.

[B42] Rabalais N. N. Turner R. E. Diaz R. J. Justic D. (2009). Global change and eutrophication of coastal waters. *ICES J. Mar. Sci.* 66 1528–1537. 10.1093/icesjms/fsp047

[B43] Rabosky D. L. Sorhannus U. (2009). Diversity dynamics of marine planktonic diatoms across the Cenozoic. *Nature* 457 183–U173. 10.1038/nature07435 19129846

[B44] Rivas R. Willems A. Subba-Rao N. S. Mateos P. F. Dazzo F. B. Kroppenstedt R. M. (2003). Description of *Devosia neptuniae* sp nov that nodulates and fixes nitrogen in symbiosis with Neptunia natans, an aquatic legume from India. *Syst. Appl. Microbiol.* 26 47–53. 10.1078/072320203322337308 12747409

[B45] Sarmento H. Gasol J. M. (2012). Use of phytoplankton-derived dissolved organic carbon by different types of bacterioplankton. *Environ. Microbiol.* 14 2348–2360. 10.1111/j.1462-2920.2012.02787.x 22639946

[B46] Selje N. Simon M. (2003). Composition and dynamics of particle-associated and free-living bacterial communities in the Weser estuary. Germany. *Aquat. Microbial Ecol.* 30 221–237. 10.3354/ame030221

[B47] Seymour J. R. Amin S. A. Raina J.-B. Stocker R. (2017). Zooming in on the phycosphere: the ecological interface for phytoplankton-bacteria relationships. *Nat. Microbiol.* 2:17065. 10.1038/nmicrobiol.2017.65 28555622

[B48] Shannon P. Markiel A. Ozier O. Baliga N. S. Wang J. T. Ramage D. (2003). Cytoscape: A software environment for integrated models of biomolecular interaction networks. *Genome Res.* 13 2498–2504. 10.1101/gr.1239303 14597658 PMC403769

[B49] Shapleigh J. P. (2006). “The denitrifying prokaryotes,” in *The Prokaryotes: Volume 2: Ecophysiology and Biochemistry* eds Dworkin M. Falkow S. Rosenberg E. Schleifer K.-H. Stackebrandt E. (New York, NY: Springer) 769–792.

[B50] Shieh W. Y. Simidu U. Maruyama Y. (1988). Nitrogen fixation by marine agar-degrading bacteria. *Microbiology* 134 1821–1825. 10.1099/00221287-134-7-1821 28551876

[B51] Suikkanen S. Laamanen M. Huttunen M. (2007). Long-term changes in summer phytoplankton communities of the open northern Baltic Sea. *Estuar. Coast. Shelf Sci.* 71 580–592. 10.1016/j.ecss.2006.09.004 18488550

[B52] Taylor J. D. Cottingham S. D. Billinge J. Cunliffe M. (2014). Seasonal microbial community dynamics correlate with phytoplankton-derived polysaccharides in surface coastal waters. *ISME J.* 8 245–248. 10.1038/ismej.2013.178 24132076 PMC3869024

[B53] Toukdarian A. E. Lidstrom M. E. (1984). DNA hybridization analysis of the nif region of two methylotrophs and molecular cloning of nif-specific DNA. *J. Bacteriol.* 157 925–930. 6321444 10.1128/jb.157.3.925-930.1984 PMC215347

[B54] Wong C. Chu K. Chen Q. Ma X. (1995). *Environmental Research in Pearl River and Coastal Areas.* Guangzhou: Guangdong Higher Education Press 1–198.

[B55] Wu J. Hong Y. Guan F. Wang Y. Tan Y. Yue W. (2016). A rapid and high-throughput microplate spectrophotometric method for field measurement of nitrate in seawater and freshwater. *Sci. Rep.* 6:20165. 10.1038/srep20165 26832984 PMC4735594

[B56] Wu M. Hong Y. Yin J. Dong J. Wang Y. (2016). Evolution of the sink and source of dissolved inorganic nitrogen with salinity as a tracer during summer in the Pearl River Estuary. *Sci. Rep.* 6:36638. 10.1038/srep36638 27833110 PMC5105154

[B57] Wu M. Song L. Ren J. Kan J. Qian P.-Y. (2004). Assessment of microbial dynamics in the Pearl River Estuary by 16S rRNA terminal restriction fragment analysis. *Continent. Shelf Res.* 24 1925–1934. 10.1016/j.csr.2004.06.016

[B58] Yu H. Yuan M. Lu W. Yang J. Dai S. Li Q. (2011). Complete genome sequence of the nitrogen-fixing and rhizosphere-associated bacterium *Pseudomonas stutzeri* strain DSM4166. *J. Bacteriol.* 193 3422–3423. 10.1128/jb.05039-11 21515765 PMC3133286

[B59] Zhang Y. Jiao N. Cottrell M. T. Kirchman D. L. (2006). Contribution of major bacterial groups to bacterial biomass production along a salinity gradient in the South China Sea. *Aquat. Microbial Ecol.* 43 233–241. 10.3354/ame043233

