# Supplementary material for: Spatial Variability and Co-acclimation of Phytoplankton and Bacterioplankton Communities in the Pearl River Estuary, China
Source: Front Microbiol. 2018 Oct 23;9:2503. doi: 10.3389/fmicb.2018.02503 (PMC6206238; doi:10.3389/fmicb.2018.02503)
Supplement: Supplementary file 1 [file Data_Sheet_1.DOCX]

**Spatial Variability and Co-acclimation of Phytoplankton and Bacterioplankton Communities in the Pearl River Estuary, China**

Jianming Zhu^1^, Yiguo Hong^2^, Sahib Zada^1^, Zhong Hu^1*^, Hui Wang^1*^

1, Biology Department, College of Science, Shantou University, Shantou, China 515063

2, School of Environmental Science and Engineering, Guangzhou University, Guangzhou, China 510006

Corresponding author: Hui Wang, [wanghui@stu.edu.cn](mailto:wanghui@stu.edu.cn), +86-754-86502721; or Zhong Hu, hzh@stu.edu.cn, +86-754-86504189

**
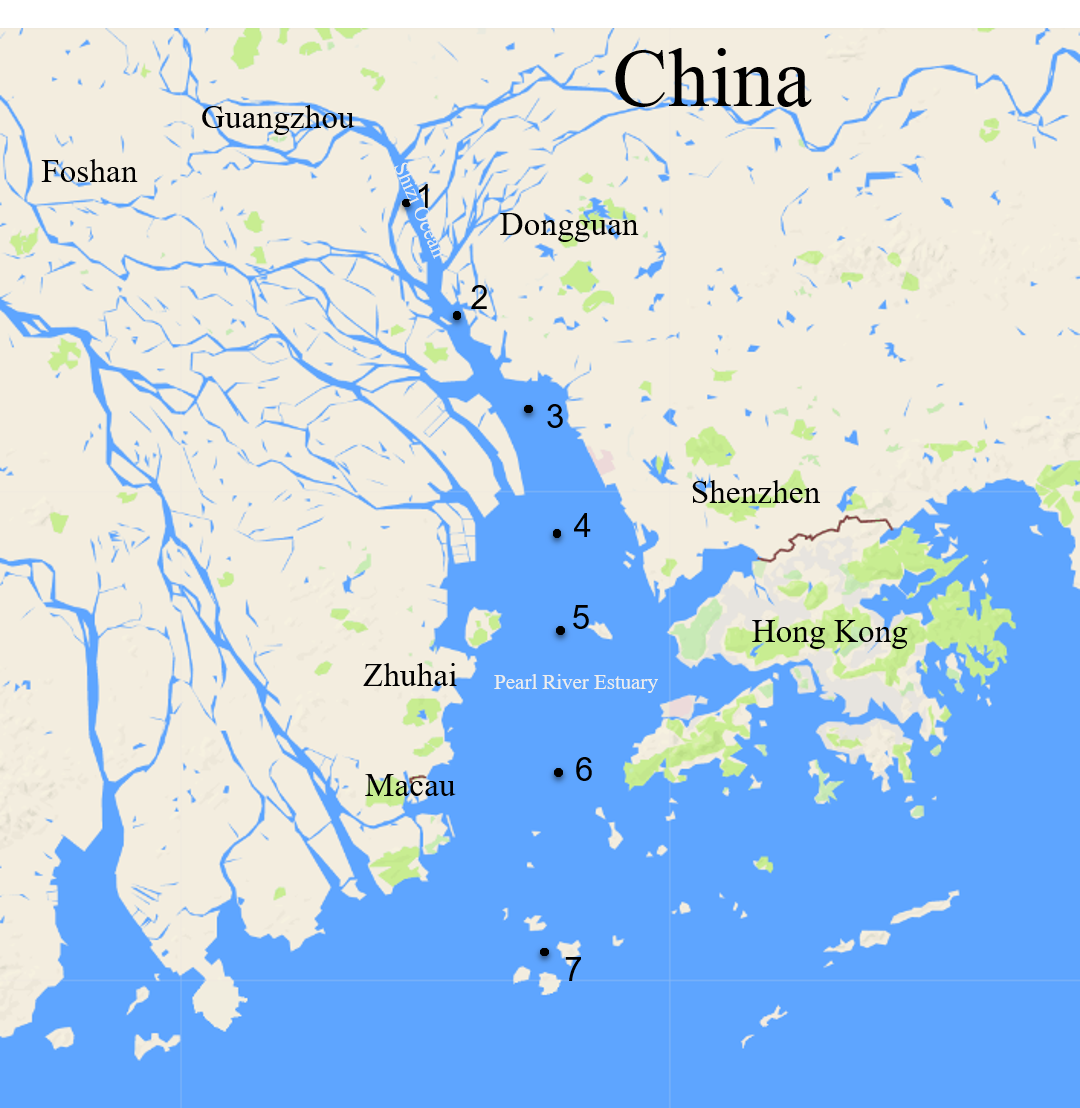
**

Supplementary Figure S1. Location of sampling sites in the PRE.


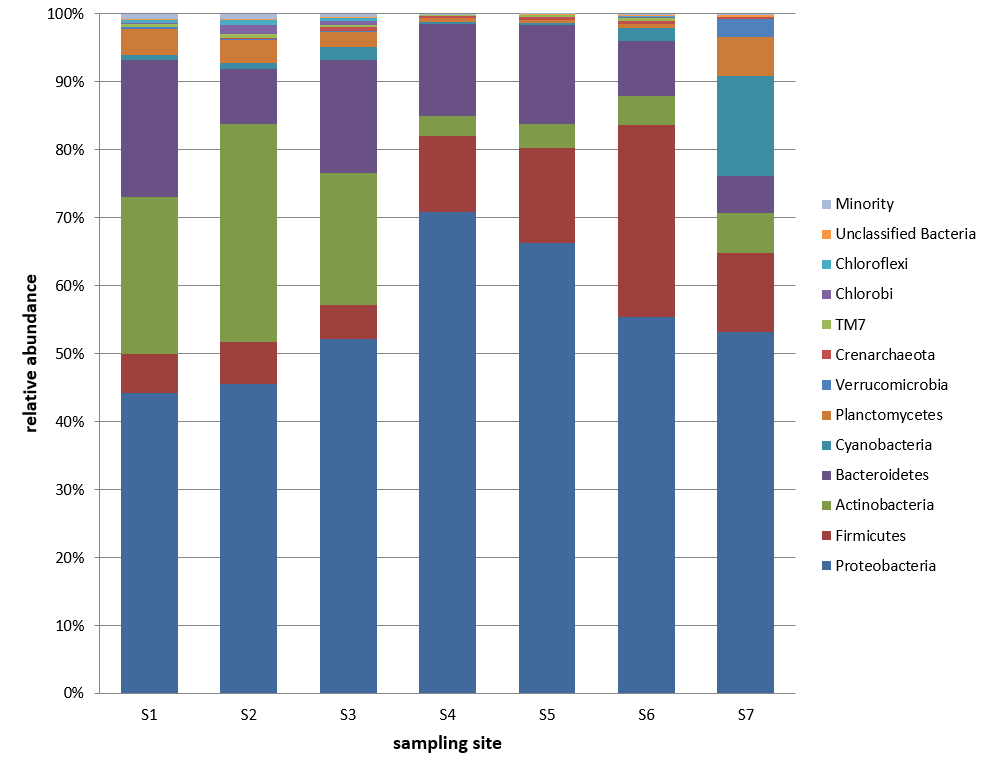


Supplementary Figure S2. Bacterioplankton composition of water samples at the phylum level in PRE. Y-axis stand for the relative abundance of bacteria-phylum in one sampling site. X-axis stand for the sampling site. (Threshold value: the sum of one site bacterial percentage more than 1%)


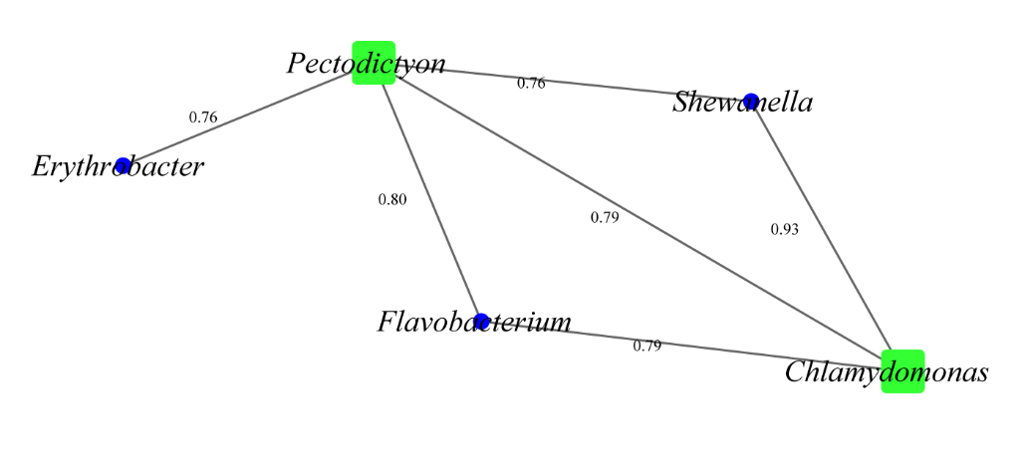


Supplementary Figure S3. IC1, the identifying cluster identified from the network (Figure 4) consisted of five units including *Flavobacterium*, *Shewanella*, *Erythrobacter*, *Chlamydomonas* and *Pectodictyon* connecting by solid grey lines. Numbers on lines represented the correlation coefficients of the nodes.


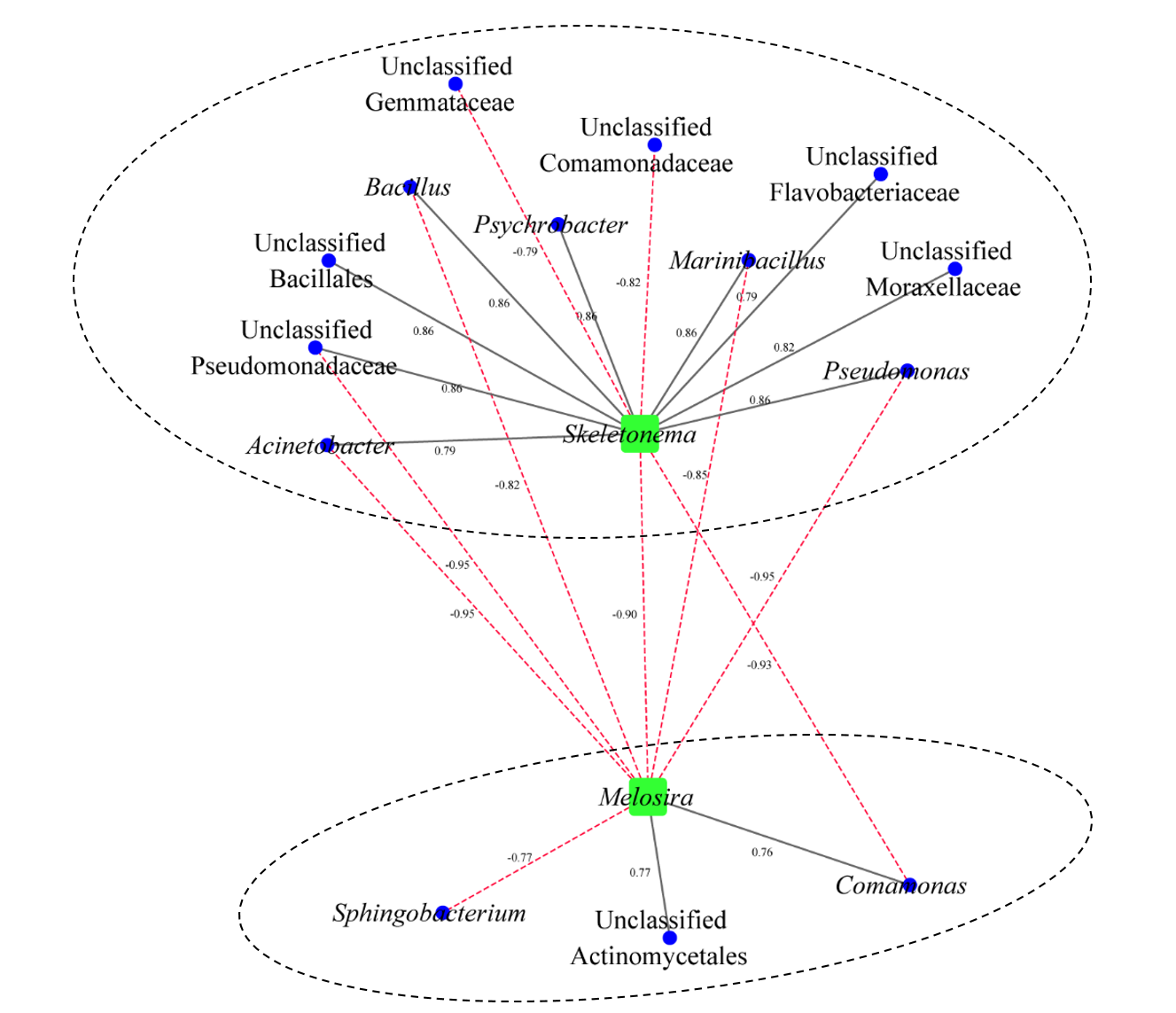


Supplementary Figure S4. The subnetwork IC2 was divided into two opposite parts by gray solid lines with the center being *Melosira* and *Skeletonema* respectively. *Melosira* shows a significant positive correlation (P <0.05) with NH_4_^+^. Numbers on lines represented the correlation coefficients of the nodes.


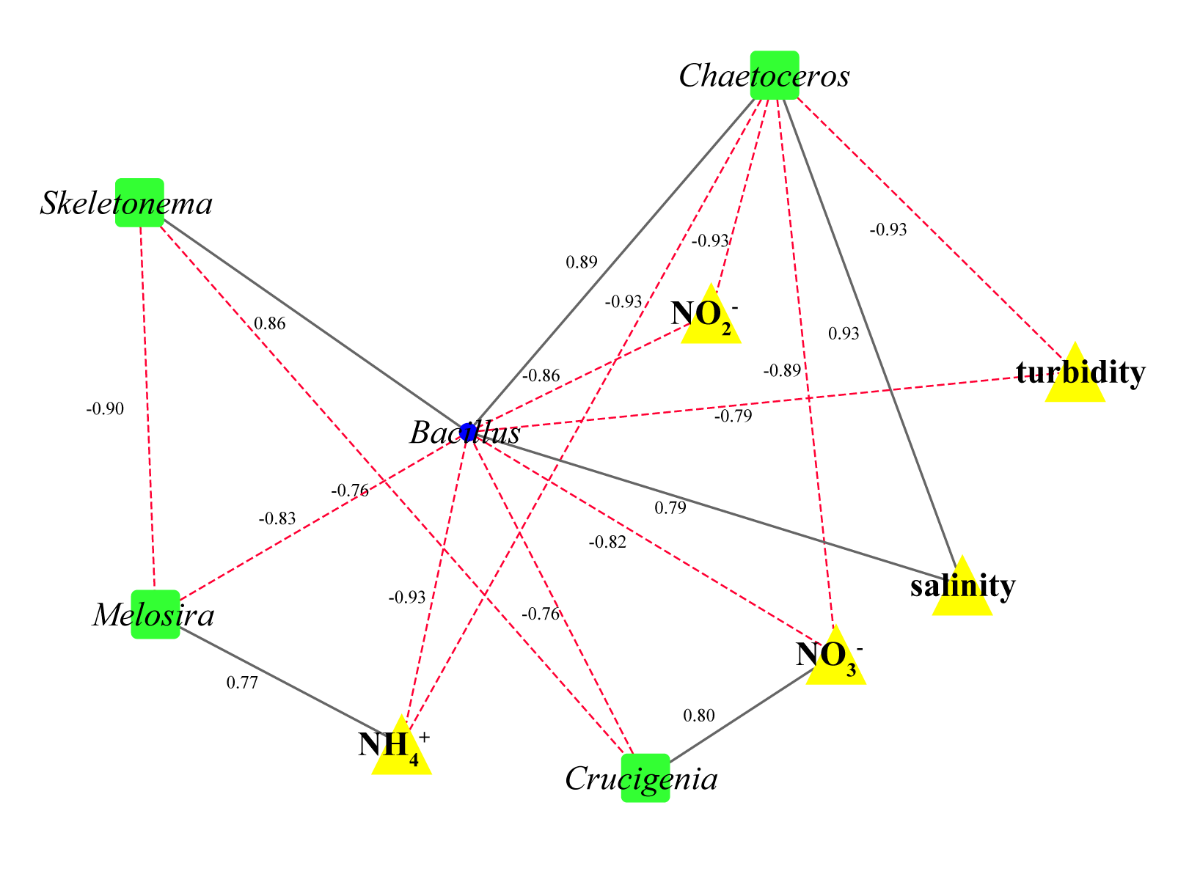


Supplementary Figure S5. IC3 shows the obvious factors that affect the tendency of *Bacillus*. Numbers on lines represented the correlation coefficients of the nodes.


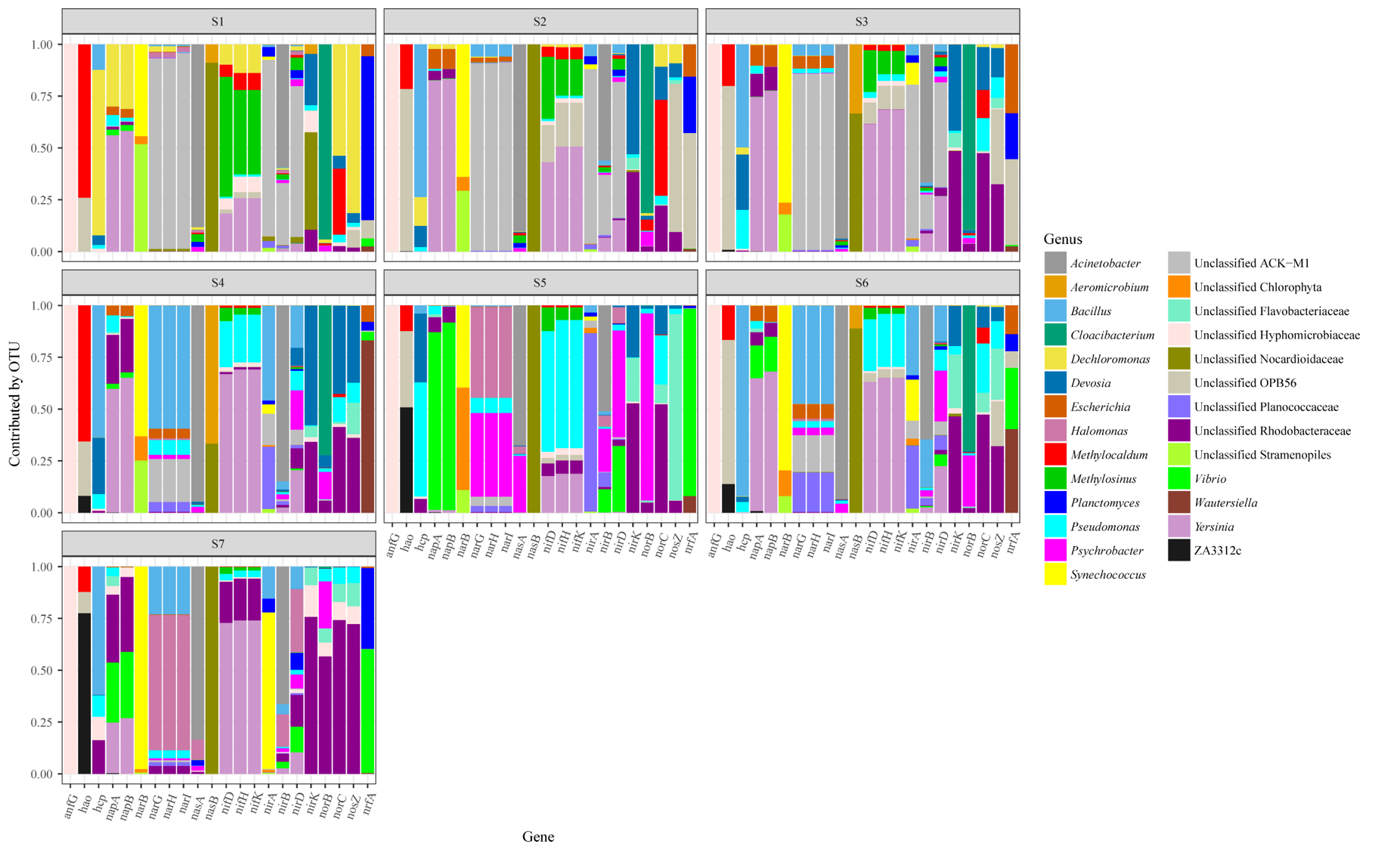


Figure S6. The dominant bacteria participated in different steps of nitrogen cycle pathway. The genes abbreviation on X axis represented the genes involved in the different steps of nitrogen cycle pathway. The bacterial OTUs contributed greatly (>20%) to any single gene were chosen to analyze their contributions in the whole process of nitrogen cycle.
